# Transjugular intrahepatic portosystemic shunt (TIPS) procedure: an assessment of the quality and readability of online information

**DOI:** 10.1186/s12911-021-01513-x

**Published:** 2021-05-05

**Authors:** Sean-Tee J. M. Lim, Martin Kelly, Logeswaran Selvarajah, Michael Murray, Timothy Scanlon

**Affiliations:** 1 Department of Surgery, Limerick University Hospital, St Nessan’s Rd, Dooradoyle, Co. Limerick, V94 F858 Ireland; 2 Department of Radiology, Limerick University Hospital, St Nessan’s Rd, Dooradoyle, Co. Limerick, V94 F858 Ireland

**Keywords:** Consumer health information, TIPS procedure, Online, Readability, JAMA, DISCERN

## Abstract

**Purpose:**

Transjugular intrahepatic portosystemic shunt (TIPS) procedure is an established procedure carried out by interventional radiologists to achieve portal decompression and to manage the complications of portal hypertension. The aim of this study was to evaluate the quality and readability of information available online for TIPS procedure.

**Methods:**

Websites were identified using the search terms “TIPS procedure”, “TIPSS procedure”, “transjugular intrahepatic portosystemic shunt procedure”, with the first 25 pages from the three most popular search engines (Google, Bing and Yahoo) being selected for evaluation with a total of 225. Each Website was grouped by authorship into one of five categories: (1) *Physician*, (2) *Academic*, (3) *For-profit*, (4) *Non-profit* (including government and public health), or (5) *Other* (discussion/social media). Readability of each Website was assessed using the Flesch-Reading Ease score, Flesch–Kincaid grade level, Gunning-Fog Index, Coleman–Liau and SMOG index. Quality was calculated using the DISCERN instrument, the Journal of the American Medical Association (JAMA) benchmark criteria and the presence of Health on the Net (HON) code certification.

**Results:**

After disregarding duplicate and non-accessible Websites a total of 81 were included. The mean DISCERN score assessing the quality of information provided by Websites was “good” (59.3 ± 10.2) with adherence to the JAMA Benchmark being 54.3%. Websites with HON-code certification were statistically significantly higher in terms of DISCERN (*p* = 0.034) and JAMA scores (*p* = 0.003) compared to HON-code negative sites. The readability scores of Websites ranged from 10 to 12th grade across calculators. Thirty-two out of the 81 Websites were targeted towards patients (39.5%), 46 towards medical professionals (56.8%) and 3 were aimed at neither (3.7%). The medical professional aimed Websites were statistically significantly more difficulty to read across all readability formulas (all *p* < 0.001).

**Conclusion:**

While quality of online information available to patients is “good”, the average readability for information on the internet for TIPS is set far above the recommended 7th-grade level. Academic Websites were of the highest quality, yet most challenging for the general public to read. These findings call for the production of high-quality and comprehensible content around TIPS procedure, where physicians can reliably direct their patients for information.

## Background

Transjugular intrahepatic portosystemic shunt (TIPS) procedure is an established procedure carried out by interventional radiologists, and is preformed to achieve portal decompression and to manage the complications of portal hypertension. The two main indications where patients have been shown to benefit from TIPS procedure include secondary prevention variceal haemorrhage and for the treatment of refractory ascites [1]. While TIPS procedure is usually well tolerated by patients, it is associated with numerous complications ranging from minor to life-threatening. Short-term complications include hepatic failure, septicaemia and abdominal cavity haemorrhage, with intermediate and long-term complications including stent stenosis and hepatic encephalopathy [2]. For patients with hepatic encephalopathy refractory to medical therapy, further intervention to reduce the shut diameter or reversing the procedure by occluding the shunt may be necessary, but is also associated with further risk [3–5]. With the chances of life-threatening complications and possible need for procedural reintervention, it is important for the patient to be well informed on the benefits and adverse outcomes associated with the procedure. The decision to perform a TIPS procedure is generally made in the scheduled setting by consultation between the patient, hepatologist and interventional radiologist, allowing time for the patient to reflect on the benefits and risks [6]. In the case of many patients, information they find on the Internet has potential to influence their healthcare-related decision-making [7]. Now more than ever, the internet has become the largest source of information worldwide for patients and doctors alike. Patients often use the internet as a source of information to research about their conditions and treatments available [8]. However, this information is unregulated and varies in quality. The popularity of health information on the internet is reflected by the increasing number of studies being published evaluating the quality of health-related information available online [9–11].

As well as displaying high-quality, reliable information, the readability of Websites must be suitable for the target patient audience. The National Institutes of Health (NIH) has suggested that patient education materials be written at a seventh-grade or lower reading level [12]. This is especially pertinent to the patient cohort with chronic liver disease who may require TIPS procedure, due to the associated lower level of formal education and poor health literacy [13, 14].

Hansberry et al. [10] have previously assesed the readability of TIPS procedure information on the Cardiovascular and Interventional Radiological Society of Europe (CIRSE) and the Society of Interventional Radiology (SIR) Websites. This limited analysis suggested that the readability of information pertaining to TIPS was considerably above the recommended level. The quality of online health information pertaining to TIPS procedure is unknown and has not been previously studied. Furthermore, an extensive evaluation of the readability of the online information pertaining to TIPS has not been previously performed. The aim of this study was to evaluate the quality and readability of information available on the Internet pertaining to TIPS procedure using recognized scoring systems. We also wished to investigate the differences between computer search engines, and the target audience of Website information, being designed either for patients or medical professionals.

## Methods

The three search terms “TIPS procedure”, “transjugular intrahepatic portosystemic shunt procedure” and “TIPSS procedure” were used for evaluation. These represented the most likely search terms to be entered by patients and medical professionals, returning 1,310,000,000, 202,000 and 33,300 results on Google search respectively. The three terms were individually entered into the three most commonly used search engines in 2020; Google, Bing and Yahoo on the 1st of June 2020, identified by Netmarketshare.com. The majority of people using the internet visit fewer than 25 Websites for a topic search, therefore the first 25 Websites displayed by each search engine result were analyzed, with 225 Websites in total [7]. All searches were conducted from the same Internet Protocol address in a cookie and cache cleared manner to reduce the influence of previous search queries.

Websites were excluded from further analysis for repetition, inaccessibility and nonrelevance. Any duplicate or overlapping Websites within and across the three search engines were discarded. The data collection methods were similar to those reported in previous studies [9, 15, 16].

Each Website was grouped by authorship into one of five categories: (1) *Physician*, (2) *Academic*, (3) *For-profit*, (4) *Non-profit* (including government and public health), or (5) *Other* (discussion/social media). The Websites were evaluated for the presence of images and videos that were relevant to TIPS procedure. Images were categorised by the type of image and information being shown. Images were also evaluated for the resolution size in pixels. Videos embedded in the Websites were evaluated for content and total runtime.

The Websites were also assessed in groups depending on if the information was either aimed at (1) patients, (2) medical professionals or (3) undetermined. This was assessed by the language being used in the articles, if it was catered to patients needs or information for doctors. Websites were classified as undetermined if it was unclear who the target audience was.

The age of each Website was recorded from the displayed latest date of information update.

The quality and reliability of the content displayed on each Webpage were assessed using three validated methods; (1) the presence or absence of the Health on Net Foundation Code (HON-code), (2) DISCERN score and (3) Journal of American Medical Association (JAMA) benchmark criteria.

The Health on the Net Foundation is a non-profit organization accredited to the United Nations, which was founded in 1995. Its aim is to protect the public from misleading health information and focuses only on human health online content. It provides HON-code certification to Websites that meet quality and reliability standards [17].

The DISCERN project was designed to judge the quality of written information on health-related Internet Websites. The DISCERN instrument is a 16 item health-care Website quality and reliability assessment tool, developed by the United Kingdom National Health Service (NHS) executive research and development programme. It provides a questionnaire to score each Website with a rating, with 80 being the highest and 16 the lowest score, indicative of a Website that is of poor quality and reliability. The first 8 questions concern the reliability of the publication and the subsequent 7 address specific details of treatment choices. The last question is an overall rating of the Website [18].

The JAMA benchmark criteria judges Websites reliability based on four components: (1) display of authorship and credentials, (2) attribution and references, (3) the date of publication and last update, and (4) disclosure of ownership, sponsorship, advertising policies, and conflicts of interest. If present on the Web page each component is awarded 1 point with a maximum of 4 [19]. The JAMA, DISCERN scores and authorship of each Website were assessed and calculated independently by 2 reviewers (S-T. L.) and (M.K.). In the case of discrepancies, a third reviewer (L.S.) was consulted to reach final consensus of calculated scores amongst all three reviewers. The interobserver variability was evaluated for both the DISCERN and JAMA score using Cohen’s kappa co-efficient (κ) as previously described [20]. Kappa is calculated by the equation κ = (Pο − Pε)/(1 − Pε), where Pο is the observed agreement among raters and Pε is the probability of agreement by chance [21]. A negative Kappa value implies that the interobserver agreement is worse than what would be expected to occur at random. A Kappa value of 0 implies that the scores have no similarity and can be explained by chance. A score of greater than 0.2 is fair and less than 0.2 is poor. Greater than 0.4 is considered to be a moderate level of agreement. A good level of agreement determined by the κ ratio is greater than 0.6. A κ value above 0.8 implies excellent agreement. When κ = 1, there is complete agreement between the observers [20].

The readability of each Website was assessed using five validated commonly used readability assessment tools: (1) the Flesch Reading Ease (FRE) score, (2) the Flesch–Kincaid grade level (FKGL), (3) the Gunning Fog Index (GFI), (4) Coleman–Liau Index (CLI) and (5) Simple Measure of Gobbledygook (SMOG) grade. The FRE score ranges from 0 to 100 with the lower the score indicating a more difficult passage to read. It is calculated on the basis of the total words, syllables, and sentences of a written passage using the following formula: 206.835 − 1.015 (total words/total sentences) − 84.6 (total syllables/total words) [22]. The FKGL score corresponds to the US reading grade level. It is calculated using the formula: 0.39 (total words/total sentences) + 11.8 (total syllables/total words) − 15.59 [23]. The GFI estimates the number of years of formal education required to read a passage of text with the higher the score indicating the difficulty of comprehension. It is calculated using the formula 0.4 [(words/sentences) + 100 (complex words/words)] [24]. The CLI score corresponds to word length and not counting of syllables like the other scores. It equates to the US reading grade level and is calculated by 5.89 × (characters/words) − 0.3 × (sentences/words) − 15.8 [25]. The SMOG grade estimates the years of education the average person needs to understand any piece of writing. The SMOG grade is calculated based on the number of polysyllabic (three or more syllables) words relative to the number of sentences [26]. All five readability scores were calculated for each Website by copying the text into a free online readability calculator tool [27].

### Statistical methods

The Shapiro–Wilk test was performed in order to analyse the distribution of the variables.

For normally distributed data ANOVA was performed with post hoc analysis using Dunnett’s T3 testing. For non-normally distributed data the Mann–Whitney U test and the Kruskal–Wallis H test were used in a bivariate test in order to search for significant differences between groups. All statistical analysis was performing on SPSS V.27. Significance was set at *p* < 0.05. Mean scores are reported in the text with standard deviation (SD) accompanying in parentheses.

## Results

**Fig. 1:**
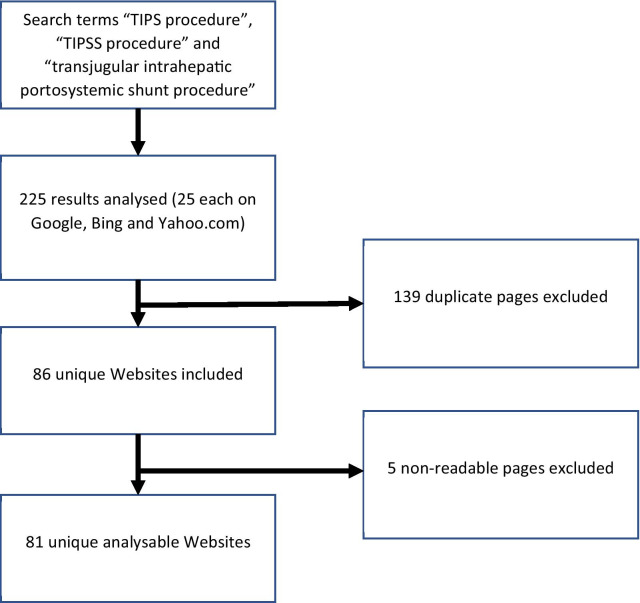
Pictorial representation of search strategy

**Fig. 2:**
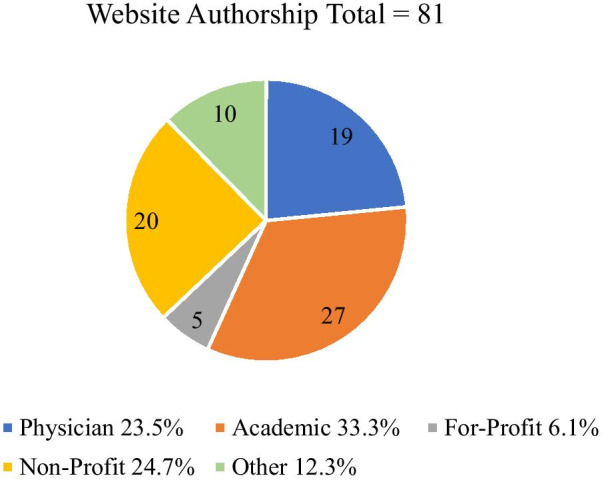
Authorship of Web sites

**Fig. 3:**
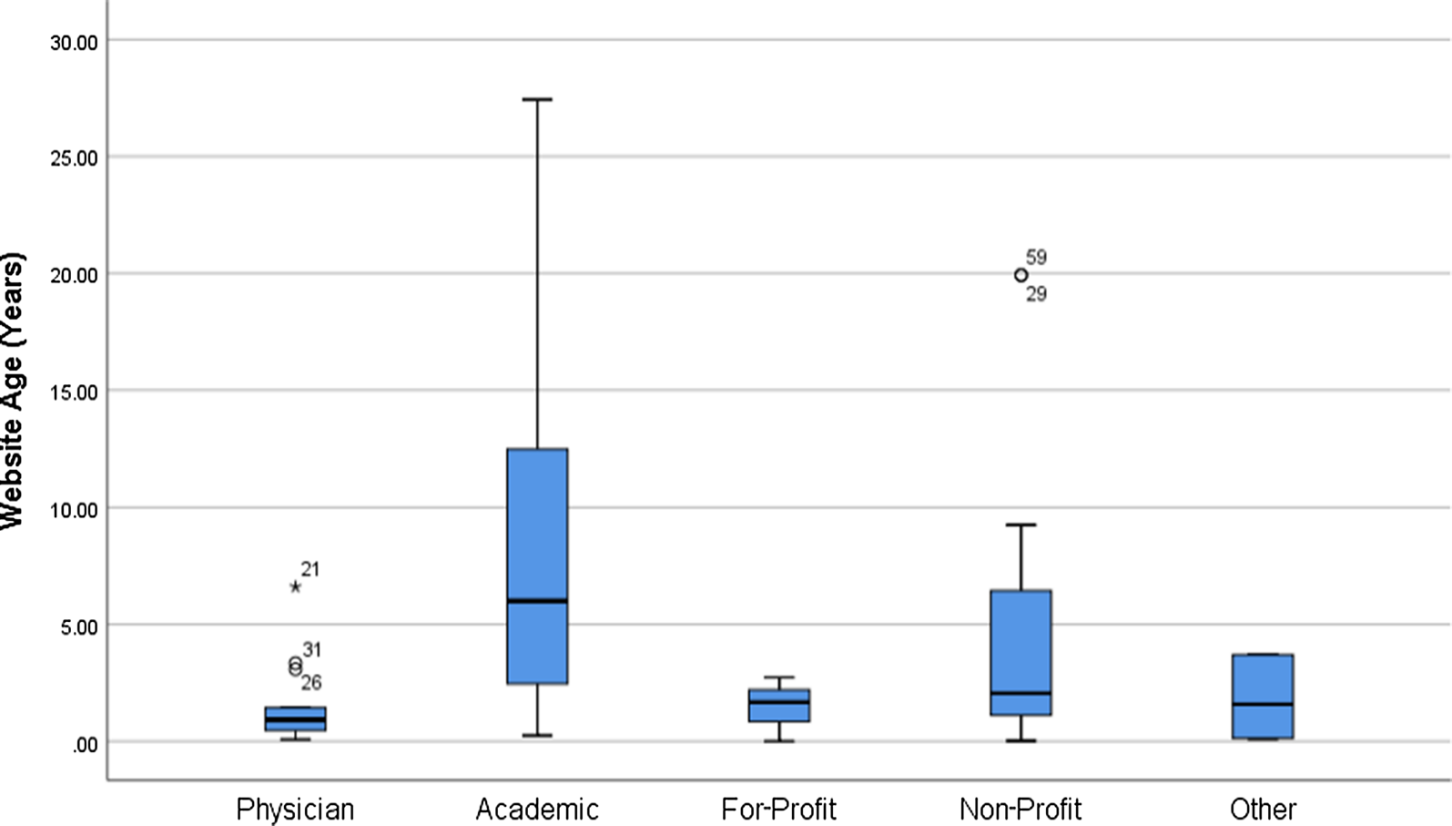
Website age by organization in years. Box plot with the vertical bar indicating range, the box indicating 25th–75th percentile, and the horizontal line indicating the median

Following the exclusion of 139 duplicate Websites, and 5 nonreadable links, 81 unique analyzable Websites were recorded (Fig. 1). The Websites were categorized according to their declared authorship (Fig. 2). Twenty-seven (33.3%) were *Academic*, 19 (23.5%) were produced by physicians who not affiliated with an academic institution, 13 (6.1%) were *For-Profit*, 20 (24.7%) were *Non-Profit* and 10 (12.3%) were classed as *Other*, being attached to discussion groups or social media sites. The average age of Websites was 5.1 years from the latest displayed update date, with the *Physician* category having the most up-to-date (1.4 years) and the *Academic* being the least up-to-date (8.2 years) (Fig. 3).

### JAMA and DISCERN

Cohen’s Kappa coefficient (κ) was calculated for both the DISCERN and the JAMA scores. It was found that the DISCERN scoring system had a ‘good’ level of interobserver agreement (κ = 0.73). The JAMA score had a higher rate of interobserver agreement which was classified as ‘excellent’ (κ = 0.85). The Shapiro–Wilk test was performed in order to analyse the distribution of the variables, and the results revealed the non-normal distribution of the values for JAMA and Discern testing. The mean JAMA benchmark criteria scores (mean ± SD = 3.1 ± 1.1)are presented in Table 1. *Academic* (3.9 ± 0.5) and *Physician* (3.5 ± 0.8) Websites scored the highest JAMA scores. There was a statistically significant difference between JAMA scores (*p* = 0.009). *Physician* Websites scored statistically higher than the other four groups (all *p* = 0.014). The *Academic* Websites scored statistically higher than the *For-Profit* (*p* = 0.001), *Non-Profit* (*p* = 0.002) and the *Other* (*p* = 0.001) categories.

**Table 1:** Summary of results

	FRE	FKGL	GFI	Coleman–Liau	SMOG	JAMA	DISCERN	Website age days
Total	44.9 ± 15.6	11.5 ± 2.8	14.6 ± 3	12.0 ± 2.3	10.8 ± 2.1	3.1 ± 1.1	59.3 ± 10.2	1876
Physician	43.7 ± 14.7	11.6 ± 2.6	14.6 ± 2.7	12.2 ± 1.8	10.8 ± 2	3.5 ± 0.8	61.1 ± 8.7	541
Academic	32.9 ± 11	13.6 ± 2.3	16.8 ± 2.3	13.6 ± 2	12.4 ± 1.6	3.9 ± 0.5	61.7 ± 8.1	3005
For-profit	51.2 ± 10.7	10.3 ± 2.1	12.4 ± 1.8	11.4 ± 1.5	9.8 ± 1.9	2.2 ± 1.3	52.2 ± 12.3	803
Non-profit	58.6 ± 9.9	9.2 ± 1.9	12.9 ± 2.9	10.3 ± 1.7	9 ± 1.3	2.6 ± 0.9	63.1 ± 9.3	1911
Other	49.5 ± 15.1	10.6 ± 2.6	13.5 ± 2.6	11.4 ± 2.9	10.1 ± 2	2 ± 1.1	45.9 ± 7.4	792
HON-code+	43.1 ± 15.9	11.9 ± 3	15.2 ± 3.1	12.1 ± 1	11.1 ± 2.2	3.7 ± 0.6	62.9 ± 8.2	1650
HON-code−	45.8 ± 15.6	11.3 ± 2.7	14.4 ± 3	12.0 ± 2.5	10.6 ± 2.1	2.9 ± 1.5	57.7 ± 10.7	2007

Overall results for each scoring system, presented as mean ± standard variation. *FRE* indicates Flesch Reading Ease, *FKGL* Flesch–Kincaid grade level, *GFI* Gunning Fog Index, *SMOG* Simple Measure of Gobbledygook, *DISCERN* DISCERN instrument, *JAMA* The Journal of the American Medical Association benchmark criteria, Website age in days

Fourty-four of the total 81 (54.3%) Webistes achieved a maximum JAMA score of 4/4 by displaying the article authorship, full references, date of update, and Website ownership.

Each Website was reviewed and scored according to the DISCERN criteria (maximum score of 80). The mean score was 59.3 (SD, 10.2; range, 35–75), equating to a website information quality grade of “good” in accordance with the DISCERN scoring bands (Table 2). The highest scoring Website was an *Academic* site (75/80). Thirteen Websites (14%) scored were classed from “poor” to “fair”, with 39 of the Websites (48.1%) regarded as excellent quality with “minimal shortcomings.” (Table 2). There was a significant difference between the five authorship categories for DISCERN overall mean scores (*p* = 0.001), with the Academic, Physician, and Non-Profit Websites having a significantly higher score than the Other category (all *p* = 0.001).

**Table 2:** DISCERN score—quality of Websites

	Percentage	Number of Websites
Very poor (16–26)	0	0
Poor (27–38)	1.2	1
Fair (39–50)	14.8	12
Good (51–62)	35.8	29
Excellent (> 62)	48.1	39
Total	100	81

### Readability

The overall mean readability scores indicated that the Websites were difficult to read (Table 1). The five readability scores were normally distributed defined by the Shapiro–Wilk test. Both the *Physician and Academic* Websites were statistically significantly more difficult to read compared to the Non-Profit category across all five readability scores (all *p* = 0.02).The mean FRE (44.9 ± 15.6) and GFI (14.6 ± 3) scores equated to “difficult to read” and “College Sophomore” reading levels respectively (22, 24). The average reading level ranged from 10 to 12th grade (SMOG 10.8 ± 2.1, FKGL 11.5 ± 2.8, Coleman–Liau 12.0 ± 2.3). Only 2.5% (2/81) of the Websites were at or below the recommended seventh-grade readability level across all readability scores. The *Academic* Websites were the most difficult to read across all five readability scores followed by *Physician* catagory. The *Non-Profit* Websites was the easiest to read across all five readability scores.

### HON-code certification

Twenty-five of the 81 Websites (30.9%) were HON-code certified. These Websites were significantly higher in quality by both DISCERN scoring (*p* = 0.034) and JAMA benchmark criteria (*p* = 0.003). The difference between the readability scores of HON-certified and non-certified Websites failed to reach significance (all *p* > 0.3).

### Search engine and target audience

The Internet search returned 70 of the same Websites across all three search engines. There were 5, 2 and 4 unique returns from Google, Bing and Yahoo respectively, producing a total of 81.When assessing for HON-code certified Websites, Google returned 27.1% (22/81), Bing 34.6% (28/81), and Yahoo 33.3% (27/81) (Table 3).

**Table 3:** Search engine and target audience

	FRE	FKGL	GFI	Coleman–Liau	SMOG	JAMA	DISCERN	Website age days
Google	44.8 ± 15.2	11.7 ± 2.8	14.9 ± 3	11.9 ± 2.1	10.8 ± 2.1	3.2 ± 0.9	61.9 ± 8.7	1629
Bing	44.3 ± 14.7	11.6 ± 2.5	14.7 ± 2.7	12.2 ± 2.3	10.8 ± 1.9	3.2 ± 1	59.8 ± 9.7	1325
Yahoo	46.7 ± 14.6	11.5 ± 2.7	14.8 ± 3	11.7 ± 2.1	10.7 ± 2	3.0 ± 1.1	60.2 ± 9.4	1521
Patient aimed	58.7 ± 9.6	9.2 ± 1.8	12.4 ± 2.5	10.3 ± 1.8	9.0 ± 1.3	2.3 ± 1.1	59.9 ± 11.5	1561
Medical professional aimed	35.3 ± 11.3	13.2 ± 2.2	16.3 ± 2.3	13.2 ± 1.9	12.1 ± 1.7	3.7 ± 0.7	59.4 ± 9.4	2156

Overall results for each scoring system, presented as mean ± standard variation. *FRE* indicates Flesch Reading Ease, *FKGL* Flesch–Kincaid grade level, *GFI* Gunning Fog Index, *SMOG* Simple Measure of Gobbledygook, *DISCERN* DISCERN instrument, *JAMA* The Journal of the American Medical Association benchmark criteria, Website age in days

Websites were classified whether they were aimed at a patient population or medical professionals (Table 3). Thirty-two out of the 81 Websites were aimed at patients (39.5%), 46 aimed at medical professionals (56.8%) and 3 were aimed at neither (3.7%). The medical professional aimed Websites were statistically significantly greater in terms of JAMA scoring compared to the patient aimed 3.7 versus 2.3, (*p* = 0.001). There was no statistically significant difference in terms of DISCERN scores (*p* = 0.825). The medical professional aimed Websites were statistically significantly more difficulty to read across all readability formulas (all *p* = 0.001).

### Website images and videos

**Fig. 4:**
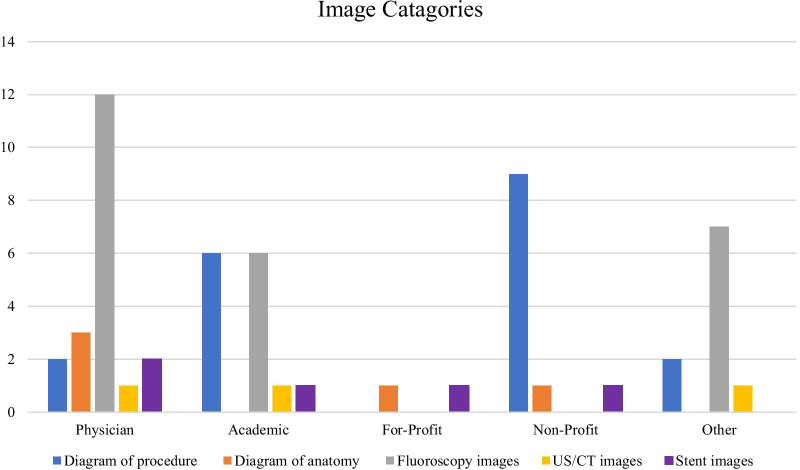
Number of images by category

In total there were 57 images displayed across the 81 Websites. The category types are represented in Fig. 4. The most common image displayed was intra procedure fluoroscopy images (25/57), followed by diagram models showing the procedure (19/57). *Physician* Websites utilized images the most with a rate of 1.1 images per Website. The *For-Profit* Websites displayed had the lowest rate at 0.4 images per Website. The *Non-Profit* Websites produced the images with the highest mean pixels (346,732 ± 35,273), with the *For-Profit* displaying the lowest (40,425 ± 4405.3). The summary of Website images is represented in Table 4. Overall there were 3 videos across all the Websites and were all located within the *Physician* category. Two of the videos discussed the procedure with real-life images with a runtime of 2:30 and 6:49 min. The third video was an illustrated representation of the procedure, lasting 2:32 min.

**Table 4:** Summary of Website images

	Total number of images	Image rate per Website	Mean image dimensions (width × length)	Mean image resolution (pixels)
Physician	20	1.1	355.9 × 323.7	128,291.8 ± 86,973.3
Academic	14	0.5	439.3 × 374.1	166,637.1 ± 56,731.8
For-profit	2	0.4	192.5 × 210	40,425 ± 4405.3
Non-profit	11	0.6	569.4 × 495.3	346,732 ± 35,273
Other	10	1	338 × 407.7	147,032.7 ± 95,596.5

## Discussion

The Internet is fast becoming the primary source of information for patients to read about their medical conditions and procedures [28]. Internet health information-seeking behavior has the possibility to improve the patient-physician relationship, allowing for the expectation of realistic treatment and outcomes [29]. In this study the Website quality for information on TIPS as assessed by JAMA and DISCERN scores varied significantly by authorship. Some reassuring findings of this study was the average DISCERN score (59.3/80) being classified as “good” in accordance with the DISCERN scoring bands. This is an improvement compared to other interventional radiology procedure online information studies with Alderson et al. [9] and Murray et al. [15] average DISCERN scores being classified as “fair”, being 49 and 47 respectively. *Academic* and *Physician* authored Websites showed the highest average quality scores, while *For-Profit* organizations and the *Other* category showed the lowest. The *Non-Profit* Websites while scoring second last in terms of JAMA, scored the highest DISCERN score. This was likely due to the majority of these Websites being comprised of patient information by hospitals and government bodies. The high DISCERN score is reflected by the patient-centric information, being concise and answering the main questions a patient may have about TIPS procedure. The poor JAMA scoring is reflected by the lack of display of authorship, date of publication and references. While it is important for Web information to be relevant and patient-focused it is also paramount that public information be transparent as to display authorship and accurate referencing, giving credibility and accountability. While the *Academic* and *Physician* Websites scored high in quality testing, they proved the most difficult to read. These two categories formed the majority of Websites at 56.8% with the easiest to read *Non-Profit* forming the minority at 24.7%. A worrying finding was the increased difficulty in readability of Websites between our study and the findings by Hansberry et al. in 2013 who previously studied the readability of TIPS procedure information on 56 CIRSE and SIR Web pages, with FRE 44.9 versus 66, FKGL 11.5 versus 8, GFI 14.6 versus 10.5, Coleman–Liau 12 versus 8.8 and SMOG 10.8 versus 10.9 respectively [10]. The average reading grade was ranging from 10 to 12th grade, far exceeding that of the suggested 7th-grade readability level for health information [12]. This is concerning as the patient cohort likely to require TIPS procedure are those with chronic liver disease, who may be more likely to have lower levels of formal education necessary to comprehend the online material [13, 30]. The age of the Web pages along with the latest date of update was evaluated in this study. Concerningly, the *Academic* Websites were the least up to date group with an average of 8.2 years. This is likely reflected by the content of scientific papers that undergo strict peer review and regulation. The most up-to-date Websites were the *Physician* authored sites. It was the findings of the authors that these sites displayed the most regular update information, this highlighting the drive of medical professionals to provide ongoing revision of their information. There was no statistically significant difference comparing these search engines in terms of Website age, readability and quality scoring. Interestingly the Websites returned by the three search engines had a large majority of overlap with 75 from Google, 72 from Bing and 74 Yahoo being included with the total of 81 being evaluated. There were 70 Website returns common to all three search engines indicating stabiltity of the results.

This is reassuring for the study results as the Websites evaluated are likely to represent the majority of online Websites searched by patients [7, 31]. A minority of Websites (30.9%) were HON-code certified. These Websites produced significantly higher in quality by both DISCERN and JAMA. On the basis of our findings, we would recommend that physicians recommend to patients the HON-code as an indicator to patients that the site is verifiable. The majority of Websites were designed for medical professionals (56.8%), with only 39.5% aimed for patients. The medical professional aimed Websites were statistically significantly greater in terms of JAMA scoring compared the patient aimed but similar for DISCERN scoring. This result may indicate that information designed for patients online can score high in terms of quality but may lack transparency in terms of authorship, date of update and detailed referencing. The medical professional aimed Websites were statistically significantly more difficulty to read compared to the patient aimed across all readability formulas with average grade score 12th–13th versus 9th–10th respectively.

Images and videos represent a visual learning tool for patients to better understand procedures and treatments [32, 33]. Our study revealed an overall low number of images per Website rate at 0.7. The majority of images were intra procedural fluoroscopy images that while may be useful to clinicians, represents little insight for the patient. The majority of beneficial diagrams of TIPS procedure were found in the *Non-Profit* category. This is reassuring as this category was mainly comprised of patient-centric information. Other images that may be beneficial to patient education are the anatomy diagrams which included illustrations of the variceal disease process. This giving potential for patients to obtain better visual insight into their condition. We acknowledge our study has a number of limitations. The health information assessment tools such as DISCERN and JAMA scoring, while being clear in their criteria for their assessment of each Website, require subjective input after screening of the Website by the assessor, and this input has the potential to lead to bias. This bias was reduced by two authors separately assessing DISCERN and JAMA scores for Websites, with a third author’s assessment being utilized if there were discrepancies. As Websites and search engines are frequently updated, the results of our study could vary from the initial search to the present time. The search was limited to the first 25 Websites displayed from each search engine, however as previously mentioned, it has been shown that the majority of internet users do not go beyond the first 25 results [7].

## Conclusion

The quality of online information pertaining to TIPS procedure “good,” as calculated using the DISCERN quality scoring instrument with JAMA benchmark adherence amongst Websites being 54.3%. The majority of Websites were not designed for patients, being aimed at medical professionals which may be irrelevant and incomprehensible. The average readability of the Websites was set at a 10th to 12th-grade reading level, far exceeding the recommended 7th-grade level. There was no statistically significant difference between the three search engines Google, Bing and Yahoo in terms of Website quality, readability and age. HON-code certification was predictive of a higher quality webpage and may be recommended to patients as the strongest predictor of high-quality online information regarding TIPS. There is a necessity for high-quality interventional radiology Websites, that are up to date, impartial, easy to read, and well-sourced. To be accessible this should be on the first 25 Websites in the main search engines.

## Data Availability

The datasets used and/or analyzed during the current study are available from the corresponding author on reasonable request.

## Abbreviations

FKGL: Flesch Kincaid Grade Level
GFI: Gunning Fog Index
CLI: Coleman–Liau Index
SMOG: Simple Measure of Gobbledygook
FRE: Flesch Reading Ease
